# Baricitinib retention rate: ‘real-life’ data from a mono-centric cohort of patients affected by rheumatoid arthritis

**DOI:** 10.3389/fmed.2023.1176613

**Published:** 2023-06-28

**Authors:** Caterina Baldi, Virginia Berlengiero, Paolo Falsetti, Alessandra Cartocci, Edoardo Conticini, Roberto D’Alessandro, Emilio D’Ignazio, Marco Bardelli, Marta Fabbroni, Luca Cantarini, Bruno Frediani, Stefano Gentileschi

**Affiliations:** ^1^Rheumatology Unit, Department of Medicine, Surgery and Neurosciences, University of Siena, Siena, Italy; ^2^Department of Medical Biotechnologies, University of Siena, Siena, Italy

**Keywords:** rheumatoid arthritis, baricitinib, retention rate, treatment, real-life, JAK inhibitors

## Abstract

**Objectives:**

The aim of this retrospective study was to evaluate baricitinib retention rate in patients affected by rheumatoid arthritis. Secondary aims were to compare the impact on treatment persistence of monotherapy and other variables such as systemic corticosteroid use, line of treatment, disease duration, sex, biomarkers positivity, and Herpes Zoster virus infection.

**Materials and methods:**

Patients with Rheumatoid Arthritis undergoing baricitinib were consecutively enrolled. Rheumatoid Arthritis diagnosis was performed with 2010 ACR/EULAR classification criteria. The cohort’s demographic, clinical and therapeutical data were retrospectively collected. The whole follow-up duration was 104 weeks.

**Results:**

Ninety-five patients affected by rheumatoid arthritis and treated with baricitinib were consecutively enrolled. At the end of follow-up, the overall retention rate was 69.3%. No statistically significant difference in retention rate was observed between patients treated with baricitinib in monotherapy or in combination with methotrexate (*p* = 0.638) while patients undergoing a steroidal treatment showed a significantly reduced treatment retention (*p* = 0.028). Contrarily, patients treated with baricitinib as a first-line b/tsDMARD showed higher drug retention (*p* = 0.002) compared to further treatment lines. Steroid employment, steroid dosage and previous treatment with bDMARDs correlated with risk of treatment discontinuation and at univariate analysis (*p* = 0.028, *p* < 0.001, and *p* = 0.002 respectively). Multivariate analysis confirmed significance for higher steroid dosage and previous treatment with bDMARDs (*p* = 0.002 and *p* = 0.046). No adverse events such as deep venous thrombosis, pulmonary embolism or tubercular infection/reactivation were reported during the study observation.

**Conclusion:**

Our data show a good baricitinib retention rate after 12 and 24 months of observation (75.1 and 69.3%, respectively). In our cohort, concomitant treatment with methotrexate did not influence treatment persistence while retention was reduced in patients undergoing a steroidal treatment and/or in multi-failure subjects.

## Introduction

Rheumatoid arthritis (RA) is a chronic, multi-systemic, immune-mediated disease that can lead to progressive joint damage, functional disability and substantial comorbidity. Early diagnosis and a prompt treatment are required to reduce clinical signs and symptoms, to prevent the development of chronic sequelae and to reduce the burden of disease from comorbidities. Since the European League Against Rheumatism (EULAR) published in 2010 its first recommendations for the management of RA with synthetic and biological disease-modifying anti-rheumatic drugs (bDMARDs), many other drugs with different mechanisms of action have been approved (1). In this regard, the recent understanding of the pivotal pathogenic role of Janus kinases (JAK) in RA lead to the development of a novel class of targeting synthetic (ts) DMARDs, the JAK inhibitors (JAKi). This class of medications represents a new cornerstone in the treatment of RA, due to both their oral route of administration and their inhibition of a large number of pro-inflammatory cytokines (2). While evidence on the efficacy and safety of baricitinib (BAR) is growing, to date, scarce data are available about BAR retention rate. The primary aim of this study was to evaluate BAR retention rate in RA patients, in a “real-life” setting. Secondary aims were to compare the impact on treatment persistence of a monotherapy approach and other variables such as the systemic use of corticosteroids (CCs), the line of treatment, disease duration, sex, Rheumatoid Factor (RF), anti-citrullinated peptide antibodies (ACPA) and HZV infections.

## Materials and methods

Demographic, clinical and therapeutic data of RA patients starting BAR treatment and consecutively referring to our arthritis center between May 2018 and July 2020 were collected. Diagnosis was performed with 2010 ACR/EULAR classification criteria for RA. Demographic, clinical and therapeutical data were collected at subject’s enrollment and at follow-up visits. We also reported data about adverse reactions to drugs including Herpes Zoster Virus (HZV) infections. The whole follow-up period was of 104 weeks. The study was conducted according to the guidelines of the declaration of Helsinki and approved by the Ethics Committee (Rhelabus 22271). Informed consent was obtained from all subjects involved in the study.

### Statistical analysis

Descriptive analysis was carried out, quantitative variables were summarized as mean ± standard deviation (SD), while qualitative ones as absolute frequencies and percentages. The association between qualitative variables and treatment interruption was evaluated with the Fisher exact test. To evaluate the mean difference between groups the t test or the non-parametric Mann–Whitney were used, according to the hypothesis of normality distribution and homoscedasticity, tested with Kolmogorov–Smirnov test and Levene’s test, respectively. Interruption rate was estimated using Cox regression and the hazard ratio (HR) and their confidence intervals were estimated. In particular univariate and stepwise multivariate Cox regression were performed. Tests were regarded to be statistically significant when *p* < 0.05. Analyses were carried out with SPSS v. 24 software.

## Results

### Descriptive analysis

Ninety-five patients affected by rheumatoid arthritis and treated with BAR were consecutively enrolled. Their demographic and clinical information at the baseline are summarized in Table 1.

**Table 1:** Values are presented as mean ± standard deviation or percentage.

	*N* = 95
Age (Years)	61.87 ± 10.12
Disease duration (months)	132.8 ± 118.9
Female (%)	67 (70.5)
RF + (%)	80 (84.2)
ACPA + (%)	55 (57.9)
History of HZV infection (%)	5 (5.3)
Corticosteroids (%)	53 (55.8)
Previous MTX (%)	94 (98.9)
Previous csDMARDs other than MTX (%)	25 (26.3)
bDMARDs naive (%)	32 (33.7)
1 previous bDMARDs failed (%)	22 (34.9)
2 previous bDMARDs failed (%)	23 (36.5)
≥3 previous bDMARDs failed (%)	18 (28.5)

Demographic, clinical and therapeutic information at the baseline of the 95 patients enrolled. ACPA, anti-citrullinated protein antibodies; bDMARDs, biologic synthetic disease modifying anti-rheumatic drugs; csDMARDs, conventional synthetic disease modifying anti-rheumatic drugs; HZV, Herpes Zoster virus; MTX, methotrexate; RF, rheumatoid factor.

BAR was given at a dosage of 2 mg in 18 (18.9%) patients and at a dosage of 4 mg in 77 (81%) patients. The mean treatment duration was 49.9 (±31.1) weeks. Forty-nine (51.6%) patients received BAR in monotherapy, while 46 (48.4%) were concomitantly receiving MTX. Fifty-three (55.8%) patients were on treatment with systemic CCs at the start of BAR at a mean dosage of 8.1 (±4.7) mg/day of prednisone or equivalent. BAR represented the first line non-conventional DMARD treatment after MTX failure in 32 (33.7%) patients. At the end of the observation time 71 (74.7%) patients were still treated with BAR. BAR was interrupted in 24 (25.3%) patients: 15 (62.5%) patients discontinued BAR because of inefficacy and 9 (37.5%) because of adverse events. Five (5.3%) patients out of 95 experienced a HZV infection while on treatment with BAR. No adverse events such as deep venous thrombosis (DVT), pulmonary embolism (PE) or tubercular infection/reactivation were reported during the study observation. Fifteen (62.5%) out of the 24 patients interrupting BAR and 38 (53.5%) out of the 71 patients continuing BAR were taking oral CCs. Thirteen (54.2%) out of the 24 patients interrupting and 33 (46.5%) out of the 71 patients continuing BAR were in combination therapy with MTX. Patients undergoing BAR as a first line agent after MTX failure were 30 (42.3%%) and 2 (8.3%) in the BAR persistence and BAR discontinuation group, respectively.

At the end of the follow-up the percentage of subjects retaining the treatment was higher in the b/tsDMARD naïve group compared to patients in which BAR represented the second or a more advanced line of treatment (*p* = 0.002). Similarly, the mean disease duration for patients that underwent BAR interruption was significantly increased when compared to patients who did not discontinue the treatment (182.9 ± 128.7 vs. 115.9 ± 111.4; *p* = 0.008). Data about patients’ sex, BAR dosages, RF and ACPA positivity, HZV infections, CCs or MTX combination therapy and history of previous bDMARDs in patients both continuing or interrupting BAR are summarized in Table 2.

**Table 2:** Data about patients’ sex, BAR dosages, RF positivity, ACPA positivity, HZV infections, CCs or MTX combination therapy and history of previous bDMARDs treatment in patients both continuing or suspending BAR.

		Ongoing BAR *N* = 71 (%)	Discontinued BAR *N* = 24 (%)	*p*-value
Sex	Female	52 (73.2)	15 (62.5)	0.437
	Male	19 (26.8)	9 (37.5)	
Baricitinib dosage	2 mg/day	11 (15.5)	7 (29.2)	0.226
	4 mg/day	60 (84.5)	17 (70.8)	
RF	Negative	9 (12.7)	6 (25)	0.196
	Positive	62 (87.3)	18 (75)	
ACPA	Negative	30 (42.3)	10 (41.7)	1.000
	Positive	41 (57.7)	14 (58.3)	
HZV while on baricitinib	No	67 (94.4)	23 (95.8)	1.000
	Yes	4 (5.6)	1 (4.2)	
Concomitant CCs	No	33 (46.5)	9 (37.5)	0.485
	Yes	38 (53.5)	15 (62.5)	
Concomitant MTX	No	38 (53.5)	11 (45.8)	0.638
	Yes	33 (46.5)	13 (54.2)	
bDMARDs naïve	No	41 (57.7)	22 (91.7)	**0.002**
	Yes	30 (42.3)	2 (8.3)	

ACPA, anti-citrullinated protein antibodies; BAR, baricitinib; bDMARDs, biologic synthetic disease modifying anti-rheumatic drugs; CCs, corticosteroids; HZV, Herpes Zoster virus; MTX, methotrexate; RF, rheumatoid factor. Statistically significant *p* values are displayed in bold.

Table 3 shows differences between subjects undergoing the two available BAR dosages. The mean age (years, 75 ± 4.91 vs. 58.81 ± 8.42) and the disease duration (months, 202.67 ± 170.79 vs. 116.52 ± 97.7) were significantly higher in the 2 mg group (*p* < 0.001 and *p* = 0.005, respectively).

**Table 3:** Demographic, clinical and therapeutic data comparison between patients treated with the two available dosages of BAR (chi-squared/*T*-test).

	Baricitinib dosage		
	2 mg/die	4 mg/die	*p*
N	18	77	
Age (years); mean (SD)	75.00 (4.91)	58.81 (8.42)	**<0.001**
Age > 65 years (%)	18 (100.0)	20 (26.0)	**<0.001**
Males (%)	8 (44.4)	20 (26.0)	0.208
Disease duration (months); mean (SD)	202.67 (170.79)	116.52 (97.70)	**0.005**
Concomitant MTX (%)	8 (44.4)	38 (49.4)	0.910
Previous bDMARD (%)	14 (77.8)	49 (63.6)	0.387
BAR treatment duration (months); mean (SD)	43.39 (32.12)	51.45 (30.91)	0.325
BAR treatment interrupted (%)	7 (38.9)	17 (22.1)	0.239
Concomitant CCs (%)	7 (38.9)	46 (59.7)	0.180
RF + (%)	16 (88.9)	64 (83.1)	0.806
ACPA + (%)	8 (44.4)	47 (61.0)	0.308
Comorbidities (%)	11 (61.1)	50 (64.9)	0.975
HSZ infection while on BAR treatment (%)	1 (5.6)	4 (5.2)	1.000
bDMARDs experienced (%)			0.057
bDMARD naïve (%)	4 (22.2)	28 (36.4)	
1 previous bDMARD failed (%)	2 (11.1)	19 (24.7)	
2 previous bDMARDs failed (%)	9 (50.0)	15 (19.5)	
≥ 3 previous bDMARDs failed (%)	3 (16.7)	15 (19.5)	

ACPA, anti-citrullinated protein antibodies; BAR, baricitinib; bDMARDs, biologic synthetic disease modifying anti-rheumatic drugs; CCs, corticosteroids; HZV, Herpes Zoster virus; MTX, methotrexate; RF, rheumatoid factor. Statistically significant *p* values are displayed in bold.

### Retention rate

**Figure 1:**
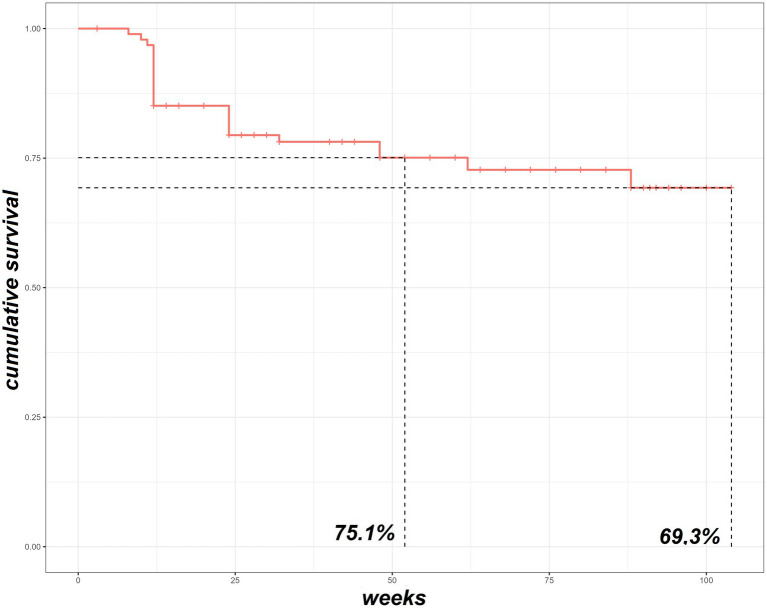
The survival Kaplan–Meier curve of our RA cohort treated with baricitinib. RA, rheumatoid arthritis.

**Figure 2:**
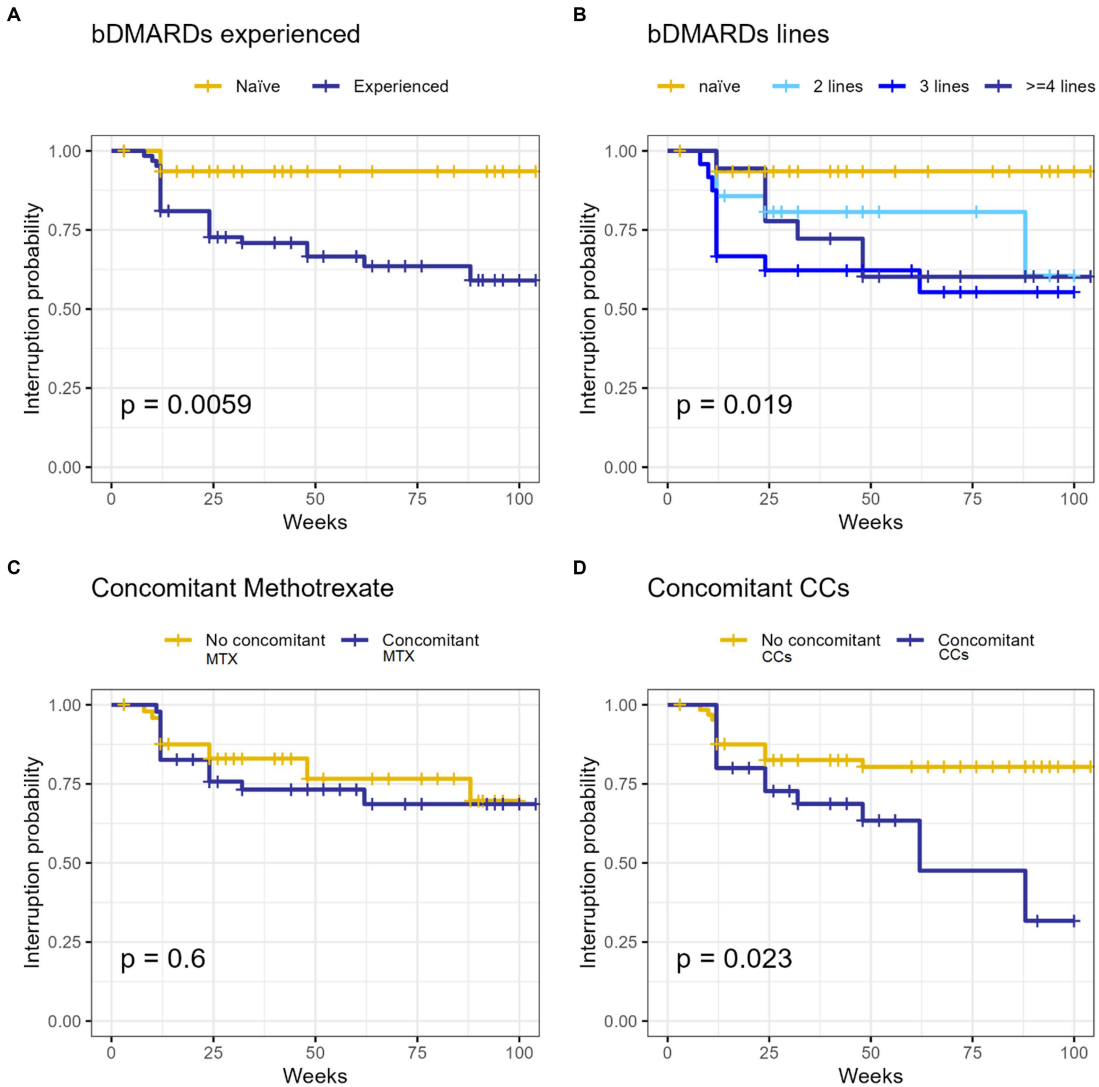
The survival Kaplan–Meier curve of our RA cohort treated with baricitinib concerning line of treatment **(A,C)** and MTX **(B)** or CCs **(D)** concomitant utilization. bDMARDs, biologic synthetic disease modifying anti-rheumatic drugs; CCs, corticosteroids; MTX, methotrexate; RA, rheumatoid arthritis.

BAR retention rate was 75.1% at 52-weeks and 69.3% at 104-week (Figure 1). No significant differences were observed between BAR retention rates in patients treated in monotherapy or in combination with MTX (*p* = 0.6). No retention rate differences were observed between patients treated with 2 or 4 mg/day (*p* = 0.226), patients with RF (*p* = 0.196) and/or ACPA (*p* = 1.000) positivity or negativity, and patients with or without HZV infection while on treatment (*p* = 1.000). On the contrary, patients who were previously exposed to bDMARDs as well as patients who were co-administered with steroids had a significantly lower BAR retention rate (*p* = 0.0059 and *p* = 0.023, respectively). Figure 2 shows survival curves stratified for concomitant drugs and line of treatment.

### Univariate and multivariate Cox analysis

Univariate analysis showed a significant correlation between BAR discontinuation and the following variables: steroid employment (*p* = 0.028), higher steroid dosage (*p* < 0.001) and previous exposure to bDMARDs (*p* = 0.017). More precisely, significance was detected for patients previously exposed to 2 (*p* = 0.008) and 3-or-more (*p* = 0.028) biological drugs. Multivariate analysis confirmed the correlation with BAR discontinuation for higher steroid dosage (*p* = 0.002) and previous bDMARDs exposure (*p* = 0.046). The output of the univariate and multivariate analysis is reported in Table 4.

**Table 4:** Univariate and multivariate Cox regression.

	Univariate analysis		Multivariate analysis	
	HR (95% CI)	*p*-value	HR (95% CI)	*p*-value
Age	1.03 (0.99–1.07)	0.197	–	–
Gender	1.66 (0.73–3.79)	0.231	–	–
Baricitinib dosage	0.52 (0.22–1.26)	0.149	–	–
RF	0.46 (0.18–1.17)	0.102	–	–
ACPA	1.05 (0.47–2.37)	0.901	–	–
HZV while on baricitinib	0.633 (0.09–4.69)	0.655	–	–
Concomitant CCs	2.48 (1.11–5.57)	**0.028**	ns	ns
CCs dosage	1.23 (1.11–1.37)	**<0.001**	1.18 (1.06–1.30)	**0.002**
Concomitant MTX	1.25 (0.56–2.79)	0.586	–	–
bDMARDs experienced	5.87 (1.38–24.96)	**0.017**	4.43 (1.03–19.18)	**0.046**
1 previous bDMARD failed	3.93 (0.76–20.26)	0.102	–	–
2 previous bDMARDs failed	7.90 (1.73–36.08)	**0.008**	ns	ns
≥3 previous bDMARDs failed	5.79 (1.20–27.90)	**0.028**	ns	ns

ACPA, anti-citrullinated protein antibodies; BAR, baricitinib; bDMARDs, biologic synthetic disease modifying anti-rheumatic drugs; CCs, corticosteroids; HZV, Herpes Zoster virus; MTX, methotrexate; RF, rheumatoid factor. Statistically significant *p* values are displayed in bold.

## Discussion

BAR is an oral JAK1/JAK2 inhibitor that can be administered, either in mono-therapy or in combination therapy with MTX, at a dosage of 2 or 4 mg once-daily in adult patients with moderate to severe active RA who are intolerant or unresponsive to one or more conventional DMARDs (3–8). In this population, the efficacy of once-daily oral BAR as mono-therapy or combination therapy was assessed in 4 randomized, double-blind phase 3 trials of 24 (RA-BUILD and RA-BEACON) or 52 (RA-BEGIN and RA-BEAM) weeks’ duration. All these four phase III studies met the ACR20 response criteria and evidenced its efficacy in reducing the health assessment questionnaire-disability index (HAQ-DI) and clinical disease activity index (CDAI) scores, while three out of four showed a significant reduction of radiographic progression measured with modified total Sharp score (mTSS) (9–12).

In this study we primarily evaluated BAR retention rate in patients affected by RA in a “real-life” setting. Results show a 104-weeks overall retention rate of 69.3%. This is in line with previous observations by other groups. Indeed, Takahashi et al. (13) and Spinelli et al. (14) found an overall BAR retention rate of 86.5% at 24 weeks and 74% at 48 weeks of observation, respectively. Furthermore, Ebina et al. (15) reported, excluding non-toxic reasons and remissions, a BAR retention rate of 72.5% at 18 weeks. In our population a high percentage of patients were concomitantly receiving MTX (48.4%) and CCs (55.8%) and were mostly treated with BAR as second-or more line of treatment (66.3%), thus suggesting a “difficult-to-treat” cohort.

In clinical practice, MTX typically represents the first-line treatment agent in patients affected by RA. By inhibiting not only IL-6 but also IL-1, matrix metalloproteinases and RF, MTX plays a beneficial role against joint destruction (16). Indeed, BAR monotherapy was inferior to BAR plus MTX in radiographic progression (4). However, accordingly to the results of a cohort study by Ebina et al. (17) we found BAR retention rate not being influenced by the concomitant MTX administration, thus suggesting that the effectiveness of this JAKi in inhibiting joint destruction may be superior in combination with MTX, although drug retention based on clinical settings may be similar compared with monotherapy. This data highlights the non-inferiority of BAR monotherapy in terms of persistence even in a “real-life” context.

Contrarily, according to our results, the concomitant use of CCs seems to negatively influence BAR retention rate as it was significantly reduced in patients receiving a systemic steroidal treatment. Moreover, univariate analysis showed a correlation between steroid co-administration and steroid dosage with the risk of treatment withdrawal. Multivariate analysis confirmed the correlation between steroid dosage and BAR discontinuation. The need of a chronic steroidal treatment usually relates with a higher activity and complexity of the disease; this could explain the reduced BAR treatment persistence in this population. In addition, the chronic use of CCs is known to increase the risk of infections, including HZV reactivation, thus leading to JAKi treatment discontinuation because of adverse events (17).

Furthermore, we observed a statistically significant reduction in treatment persistence in patients with a longest disease duration that could also be related to a history of drug multi-failure owing to a resistant disease. In opposition to what Ebina et al. reported, we did not find any statistically significant differences in BAR retention rates between males and females (17). Similarly, in our cohort, RF and/or ACPA positivity did not influence BAR persistence.

Another concern is whether the prior administration of bDMARDs may affect the drug persistence of BAR treatment. We observed a statistically reduced BAR retention rate between bDMARDs naïve and non-naïve patients, with the latter having a reduced BAR persistence. This could be related to the stronger treatment resistance and to a more complex and recalcitrant type of disease. However, in our cohort, univariate analysis showed a correlation between BAR withdrawal and patients already treated with 2 or ≥ 3 bDMARDs while no significance emerged when considering subjects previously administered with only one bDMARD. These findings are in line with some previous evidence showing BAR to be effective in multiple bDMARDs refractory patients, with the prior use of bDMARDs not affecting its clinical efficacy (13, 14, 18, 19).

In 2022, the Oral Rheumatoid Arthritis Trial (ORAL surveillance study) found a higher risk of major adverse cardiovascular (CV) events and venous thromboembolic events (TVE) in patients with RA and CV risk factors treated with Xeljanz (tofacitinib) than with Tumor Necrosis Factor inhibitors (TNFi) (20). Similarly, the preliminary findings from an observational study involving BAR suggest an increased risk of these adverse events in RA patients treated with BAR compared with those treated with TNFi (21). Based on these data, the European Medicines Agency and the Pharmacovigilance Risk Assessment Committee have recently endorsed the measures to minimize the risk of JAKi related serious side effects (22). Consequently, the 2022 updated version of EULAR recommendations on RA suggests that an age over 65 years, a history of current or past smoking, the presence of risk factors for CV events, malignancy, and TVE must be taken into account when considering to prescribe a JAKi (23).

However, in our cohort of patients no major adverse CV events were observed. Moreover, none of the patients experienced episodes of DVT/PE or tuberculosis infection, thus confirming once more the good BAR safety profile (24). Our results show a satisfactory long-term retention rate of BAR. Moreover, we observed that the concomitant treatment with MTX does not negatively influence BAR treatment persistence. Contrarily, BAR retention rates were reduced in patients undergoing a systemic steroidal treatment and in bDMARDs multi-failure patients, maybe due to a stronger treatment resistance and to a more complex and recalcitrant type of disease.

This study has some limitations, including the absence of a control group and the lack of data about disease activity. Moreover, the small sample size may negatively affect the detection of less common adverse events such as the occurrence of cancer or thromboembolic diseases.

Finally, regarding infectious adverse events, BAR safety profile showed to be satisfactory, thus confirming already available data. Further and larger studies are needed to confirm those results, especially concerning cardiovascular and neoplastic adverse events. These novel findings may provide new insight for the management of BAR treatment in clinical practice.

## Data availability statement

The raw data supporting the conclusions of this article will be made available by the authors, without undue reservation.

## Ethics statement

The studies involving human participants were reviewed and approved by the Azienda Ospedaliero Universitaria Senese. The patients/participants provided their written informed consent to participate in this study.

## Author contributions

CB and SG designed the study and drafted the manuscript. AC performed the statistical analysis. VB, PF, EC, RD’A, ED’I, MB, MF, LC, and BF collected the data for this study in the final dataset. BF, LC, and SG revised the draft of the manuscript. All authors contributed to the article and approved the submitted version.

## Conflict of interest

The authors declare that the research was conducted in the absence of any commercial or financial relationships that could be construed as a potential conflict of interest.

## Publisher’s note

All claims expressed in this article are solely those of the authors and do not necessarily represent those of their affiliated organizations, or those of the publisher, the editors and the reviewers. Any product that may be evaluated in this article, or claim that may be made by its manufacturer, is not guaranteed or endorsed by the publisher.
